# Circular RNA circCCDC66 promotes glioma proliferation by acting as a ceRNA for miR-320a to regulate FOXM1 expression

**DOI:** 10.18632/aging.203258

**Published:** 2021-07-12

**Authors:** Ling Qi, Weiyao Wang, Guifang Zhao, Hong Jiang, Yu Zhang, Donghai Zhao, Hong Jin, Haiyang Xu, Hongquan Yu

**Affiliations:** 1 The Sixth Affiliated Hospital of Guangzhou Medical University, Qingyuan People's Hospital, Qingyuan 511518, Guangdong, China; 2 Department of Pathophysiology, Jilin Medical University, Jilin 132013, Jilin, China; 3 Department of Pathology, Jilin Medical University, Jilin 132013, Jilin, China; 4 Department of Ophthalmology, China-Japan Union Hospital of Jilin University, Changchun 130033, Jilin, China; 5 Department of Neurovascular, First Hospital of Jilin University, Changchun 130021, Jilin, China; 6 Department of Oncological Neurosurgery, First Hospital of Jilin University, Changchun 130021, Jilin, China

**Keywords:** glioma, FOXM1, circRNA, circCCDC66, miR-320a

## Abstract

Background: In this study, we determine the potential roles and uncover the regulatory mechanisms of circCCDC66 in regulating cell growth and cell metastasis of glioma.

Methods: qRT-PCR was used to detect the expressions of circCCDC66 in gliomas and tissues. The biological function of circCCDC66 in glioma cell lines was elucidated by functional experiments. Cell counting kit-8 and transwell were used to detect the effect of circCCDC66 on the proliferation, migration and invasion of glioma cells. Bioinformatics analysis was applied to reveal the targets of circCCDC66.

Results: The results showed circCCDC66 was overexpressed in glioma and acted as an oncogene. CircCCDC66 knockdown suppressed the proliferation, migration, and invasion of glioma cells. We constructed a circCCDC66 regulating miRNA network and revealed miR-320a was a potential target of circCCDC66, which was down-regulated in high-grade gliomas compared to low-grade gliomas. Bioinformatics analysis showed circCCDC66-miR-320a/b axis was involved in regulating multiple cancer-related pathways. Furthermore, we identified FOXM1 as a key target of circCCDC66, which was involved in regulating DNA damage response pathways. In mechanism study, circCCDC66 could sponge miR-320a, thereby increasing the expression of FOXM1.

Conclusions: CircCCDC66 could facilitate glioma cells proliferation, invasion and migration by down-regulating miR-320a and up-regulating FOXM1.

## INTRODUCTION

Glioma, a widely occurring carcinoma, focuses on the central nervous system [1]. It can be divided into astrocytoma, ependymal tumor, and oligodendroglioma [2]. According to epidemiological surveys, the incidence of gliomas has increased significantly over the past 20 years. Due to its high morbidity and high variability, no specific strategies have been developed [3]. It is therefore urgently needed to develop new effective therapeutic targets to hinder the development of glioma [4].

Circular RNAs (circRNAs), belonging to non-coding RNAs with unknown functions, are widely expressed in many species. They have covalent and closed-loop structures. CircRNAs are considered to be the major epigenetic regulators in the pathogenesis of various diseases and are identified to display important roles in tumorigenesis and development [5]. For example, circRNA_102171 interacted with CTNNBIP1, and prevented it from interacting with β-catenin/TCF3/TCF4/LEF1 complex, resulting in activation of the Wnt/β-catenin pathway, thereby promoting the progression of papillary thyroid cancer [6]. Hsa_circ_100395 has been shown to regulate TCF21 through miR-1228 to inhibit the malignant behavior of lung cancer, providing a new possible mechanism for the progression of lung cancer [7].

In recent years, the rapid development of sequencing technology has provided important technical support for tumor research to find new molecular targets [8–11]. For gliomas, studies have shown that circCCDC66, a circRNA derived from unknown molecular functional genes, is expressed in glioma tissues more than three times higher than normal tissue samples obtained [12]. It has been reported that circCCDC66 is up-regulated in colon cancer and has a negative correlation with prognosis [13]. In this project, we found that circCCDC66 was over-expressed and that the expression of FOXM1 was increased by sponge miR-320a, which promoted the progression of glioma cells. Our research data suggest that circCCDC66 perhaps exhibits important roles in the progress of gliomas.

## MATERIALS AND METHODS

### Samples

12 pairs of glioma samples and corresponding nearby control of surgical resection of patients ranging from January 2012 to January 2014 were derived from our hospital. All samples were preserved at -80° C until RNA extraction. Our experiments were approved by the Ethics Committee of our hospital, and all the subjects were unanimously signed informed consent.

### Cell lines

Normal human astrocyte cell (HEB) and human glioma cells (U87, SW1783 and U373) were derived from ATCC or our lab. HEB U87 and U373 cells were cultured in DMEM medium (Gibco, USA) containing 10% FBS (TransGen Biotech, China). SW1783 cells were cultured in RPMI-1640 (Hyclone, USA) medium supplied with 10% FBS (TransGen Biotech, China). All the indicated cells were under a 37° C incubator containing 5% CO_2_.

### RNA extraction and quantification

Trizol (Takara, China) was used to extract total RNA from cells and tissues as described in the manual. Reverse transcription system was performed as follows: 10 μL volume included 1mg RNA with TransScript First-Strand cDNA Synthesis SuperMix (TransGen Biotech, China), followed by subjecting to qRT-PCR with indicated primers via TransStart Green qPCR SuperMix (TransGen Biotech) on Applied Biosystems 7500 System (Applied Biosystems, USA). We finally calculated relative RNA expression by the 2^−ΔΔCt^ method.

### Cell transfection

SiRNAs targeting circCCDC66 were synthesized by GenePharma (Shanghai, China). MiRNA mimics, inhibitor, mimics NC, inhibitor NC, and siRNAs were provided by GenePharma. pcDNA3.1 circRNA mini construction comprising circCCDC66 was a highly expressed cassette, and pcDNA3.1 including FOXM1 indicated over-expressed plasmid. The above-mentioned plasmids were separately transfected into indicated cells by Lipofectamine 3000 (Invitrogen) as manual instructions. The sequence was as follows: si-circCCDC66, CAAUUAGAGCAUCAGGAAA; si-NC, UUCUCCGAACGUGUCACGUTT.

### CCK-8 assay

Cell proliferation assay was conducted by Cell Counting Kit (Dojindo, Japan) at 24 h post-transfection. 2000 cells were re-seeded in 96-well per well and then added 10ul of CCK-8 solution at the indicated time for 2 h incubation. Cell proliferation was measured at specified days. Absorbance value in 450 nm was detected by the microplate reader (BioTek Instruments, USA) [14].

### Transwell assay

8μm pores of transwell chamber (Costar, USA) with 8.0 mm pore size polycarbonate membrane (Corning, USA) and Matrigel film (BD Biosciences) for coating upper chambers were applied to conduct invasion assay. At the same time, the upper chamber was a pre-coated membrane without matrix gel. The cells were placed in the upper chamber for the migration assay. The treatment chamber including 200μl of medium absence serum and lower chamber with 600μl of medium containing 10% FBS had seeded 1×10^5^ cells as indicated at 37° C with 5% CO_2_ overnight. On the following day, non-immigrated or non-invasive cells on the top side were eliminated by cotton swabs. The insert was fixed by methanol for 20 minutes and then dyed by DAPI at the concentration of 10ug/mL for 15 minutes. We counted migrated or invade cells to the membrane bottom and captured images by microscope and calculating the number of cells from three independent experiments in triplicate.

### Luciferase assay

A circular RNA Interactome database was applied to predict the downstream target of miRNA. The circCCDC66 sequence or the wild-type and mutant binding sites of miR-320a or miR-320a in FOXM1 3'UTR were subcloned into pmirGLO dual-luciferase reporter to construct circCCDC66-Wt/Mut and FOXM1-Wt/Mut, and co-transfect the plasmid with miR-320a mimic or miR-320a mimic into SW1783 or U373 cells. The association between circCCDC66/ FOXM1 3’-UTR or miR-320a was detected by luciferase assay. Relative luciferase activity was determined by dual-luciferase reporter assay (Promega Corporation, USA) [15]. The luciferase reporter analysis required a biological triple repeat.

### Statistical analysis

The representative data were shown as mean ± SD. All the data was calculated after three independent experiments, followed by SPSS 16.0 software analysis (IBM, USA). The difference that existed in two comparison groups or multiple groups in indicated experiments was determined by Student's t-test. The differences between tumor and normal tissues were counted by paired samples t-test. Correlation between circCCDC66 and clinical-pathological profile was determined by Fisher's exact test [16–20]. The log-rank test was applied to inspect the difference between highly and lowly expressed circCCDC66 groups. The obvious difference was indicated as the *P*-value was less than 0.05 [21–23].

## RESULTS

### Enhanced circCCDC66 in promoted glioma cell proliferation

**Figure 1:**
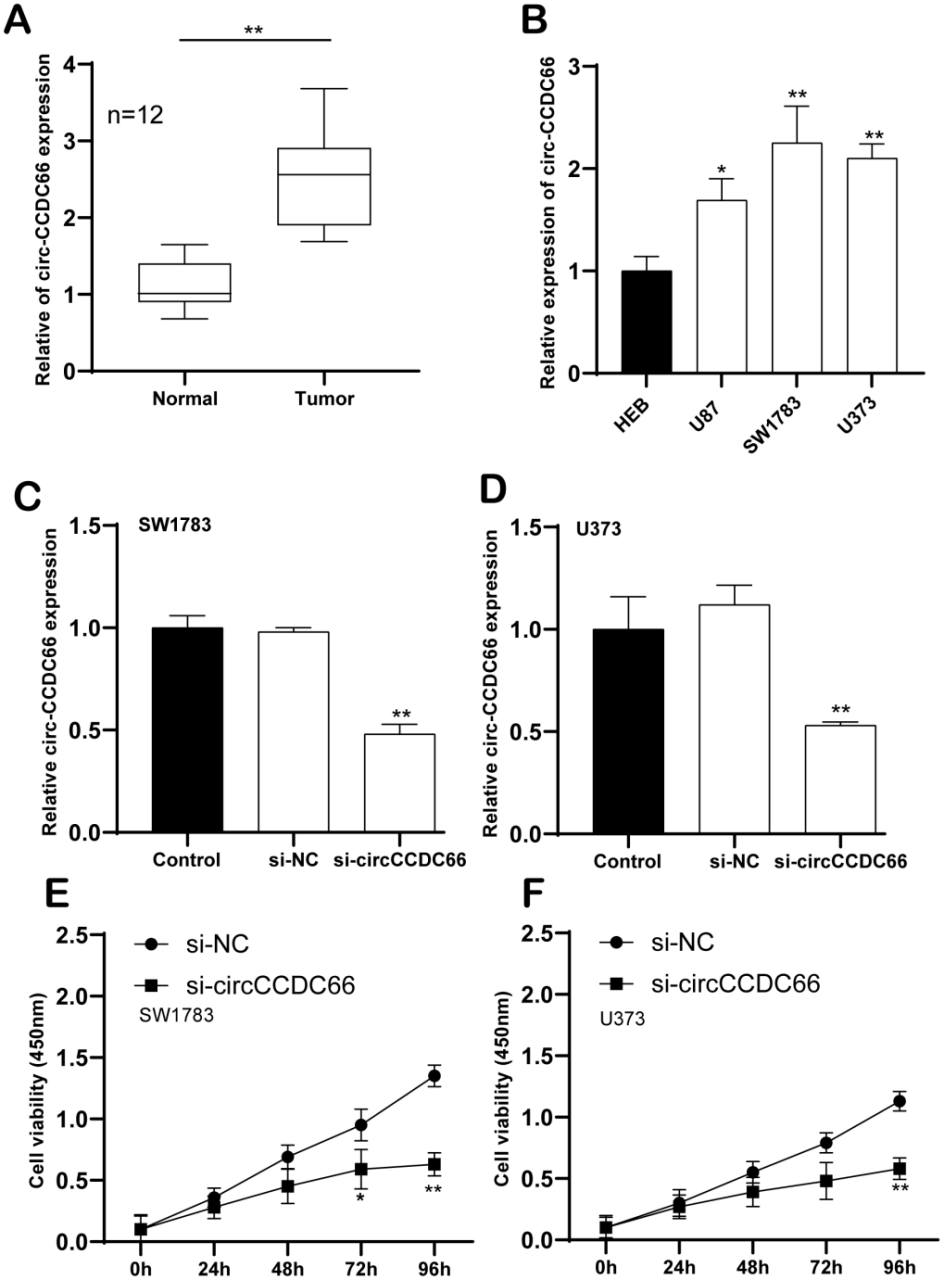
**Enhanced circCCDC66 in promoted glioma cell proliferation.** (**A**) Relative expression of circCCDC66 in glioma tissue samples and their paired non-cancerous tissue samples measured by RT-qPCR. (**B**) Relative expression of circCCDC66 in glioma cell lines and normal cell line measured by qRT-PCR. (**C**, **D**) Relative circCCDC66 expression in SW1783 and U373 cells after transfection. (**E**, **F**) Cell viability was decreased in SW1783 and U373 cells transfected with si-circCCDC66. *P < 0.05, **P < 0.01, ***P < 0.001.

As far as we know, circCCDC66 has not been reported in gliomas. This study aims to explore whether circCCDC66 affects gliomas and their mechanisms. To begin our research, we used quantitative real-time polymerase chain reaction to detect the expression of circCCDC66. qRT-PCR data demonstrated circCCDC66 level was increased in glioma samples compared to that in the normal group (Figure 1A). A similar result was observed in glioma cell lines. Compare to a normal cell, the most heightened level of circCCDC66 was shown in SW1783, while the lowest level of that was exhibited in U87 (Figure 1B). Our data suggested that circCCDC666 perhaps exhibited considerable function during the cancerous process of glioma. Then, we performed a functional analysis to study circCCDC66 in glioma cells. SiRNAs specifically targeting circCCDC66 were used in this part. Our data illustrated siRNA demonstrated strong efficiency in SW1783 and U373 cells (Figure 1C, 1D). CCK-8 assay displayed suppressed circCCDC66 retarded both SW1783 and U373 cell growth (Figure 1E, 1F).

### Cell migration and invasion were promoted by circCCDC66

**Figure 2:**
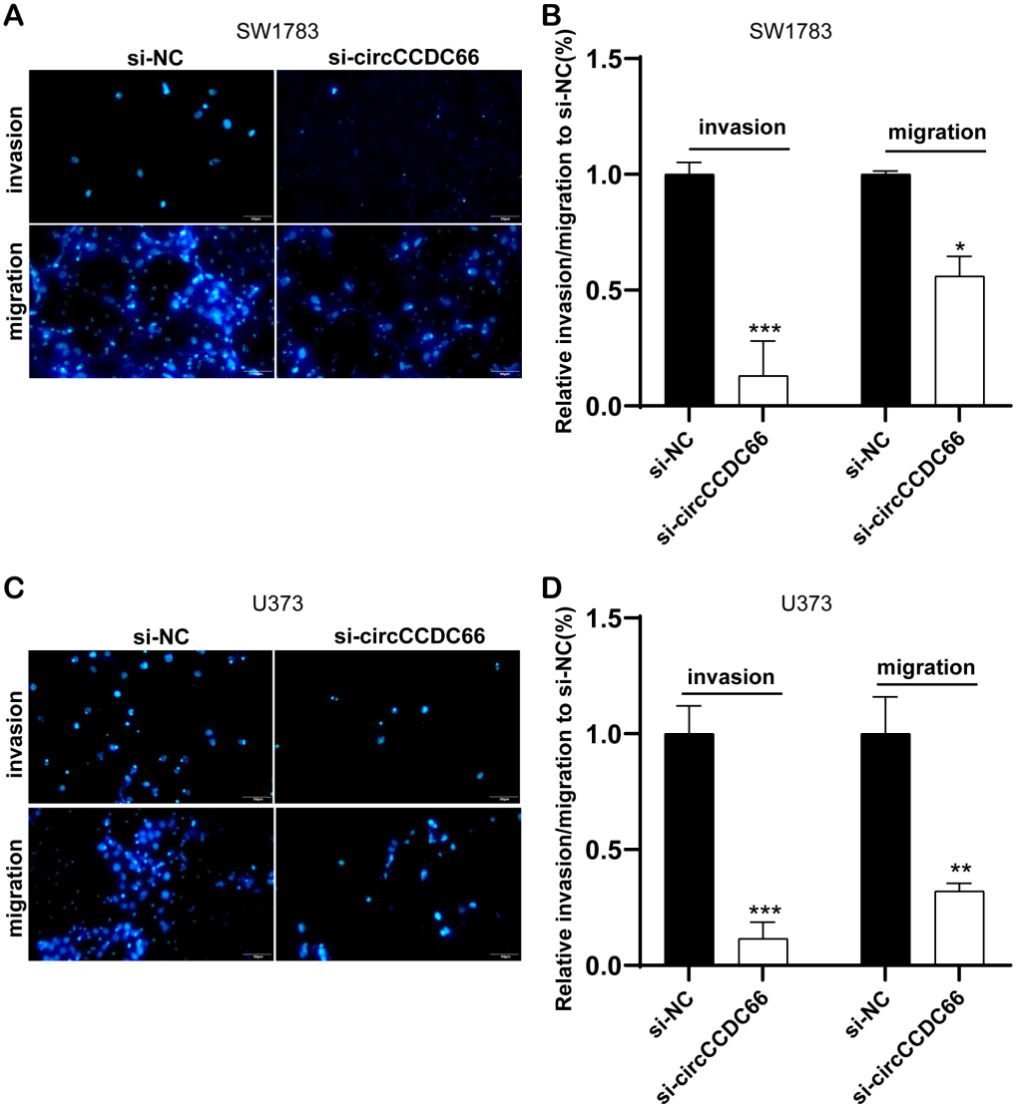
**Cell migration and invasion were promoted by circCCDC66.** (**A**, **B**) Transwell assays were used to detect cell migration and invasion capacities of SW1783 cells after transfection. (**C**, **D**) Transwell assays were used to evaluate cell metastasis potential after transfection in U373 cells. *P < 0.05, **P < 0.01, ***P < 0.001.

Furthermore, cell migration and invasion were then detected using transwell migration and invasion assay. Our results showed that the knockdown of circCCDC66 significantly suppressed cell metastasis in both SW1783 and U373 cells. The migrated and invaded SW1783 cells were remarkably reduced by 83% and 47% after silencing circCCDC66 compared to the control group (Figure 2A, 2B). Meanwhile, the migrated and invaded U373 cells were remarkably reduced by 91% and 75% after silencing circCCDC66 compared to the control group (Figure 2C, 2D).

### Construction of circCCDC66-miRNA interacting network

**Figure 3:**
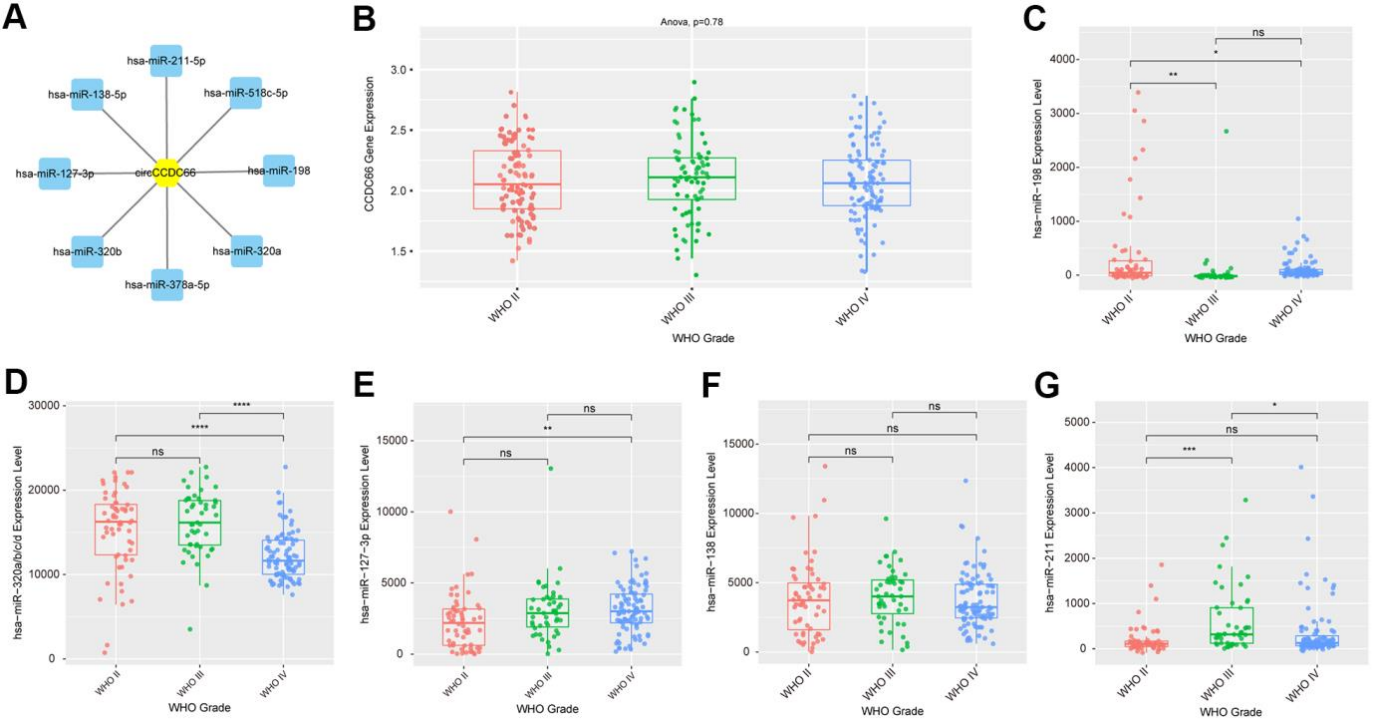
**Construction of circCCDC66-miRNA interacting network.** (**A**) CircCCDC66 may interact with 8 miRNAs using RegRNA 2.0 database. (**B**) The expression level of circCCDC66 in different WHO Grade. (**C**–**G**) The expression level of miRNA that circCCDC66 may interact with in different WHO Grade.

To predict the binding miRNAs of circCCDC66 in gliomas, we applied bioinformatics analysis using RegRNA 2.0 database. Eight miRNAs, including hsa-miR-320a, hsa-miR-518c-5p, hsa-miR-320b, hsa-miR-198, hsa-miR-211-5p, hsa-miR-127-3p, hsa-miR-378a-5p, hsa-miR-138-5p, were identified as the potential targets of circCCDC66. In order to analyze the expression pattern of these miRNAs in glioma samples, we used CGGA database. As presented in Figure 3, we found that Compared with normal samples, circCCDC66 in gliomas was significantly up-regulated. However, circCCDC66 had no significant difference in different WHO grades. hsa-miR-198 was down-regulated in WHO III/IV glioma samples compared to WHO II glioma samples. MiR-320a/b was down-regulated in WHO IV glioma samples compared to WHO II/III glioma samples. However, we found hsa-miR-127-3p and hsa-miR-211-5p were up-regulated in advanced stage glioma samples. The present study focused on hsa-miR-320a/b, which had been reported to be a tumor suppressor in cancers.

### Bioinformatics analysis of circCCDC66-miR-320a/b axis

Then, we constructed a circCCDC66-miR-320a/b-mRNAs network in gliomas using Starbase datasets, which was showed in Supplementary Figure 1A. A total of 2142 targets of miR-320a and 1882 targets of miR-320b were included in this network. Meanwhile, 1864 targets were identified to be targets of both miRNAs (Supplementary Figure 1B). The interaction among these proteins was also revealed using PPI network analysis (Supplementary Figure 1C). GO analysis showed circCCDC66 was involved in regulating Protein metabolism, Regulation of nucleobase, nucleoside, nucleotide, and nucleic acid metabolism, RNA metabolism, Regulation of gene expression, epigenetic, Regulation of translation, Protein targeting, Mitosis, Morphogenesis (Supplementary Figure 1D). KEGG pathway analysis demonstrated that circCCDC66 was related to the regulation of TRAIL signaling pathway, IGF1 pathway, signaling events mediated by Hepatocyte Growth Factor Receptor (c-Met), PDGF receptor signaling network, signaling events mediated by VEGFR1 and VEGFR2, VEGF and VEGFR signaling network, EGFR-dependent Endothelin signaling events, Sphingosine 1-phosphate (S1P) pathway, Proteoglycan syndecan-mediated signaling events (Supplementary Figure 1E).

### Identification of key targets of circCCDC66-miR-320a/b axis

Then, we aimed to identify key targets of the circCCDC66-miR-320a/b axis using Mcode analysis in Cytoscape software. Three key PPI networks were identified.

**Figure 4:**
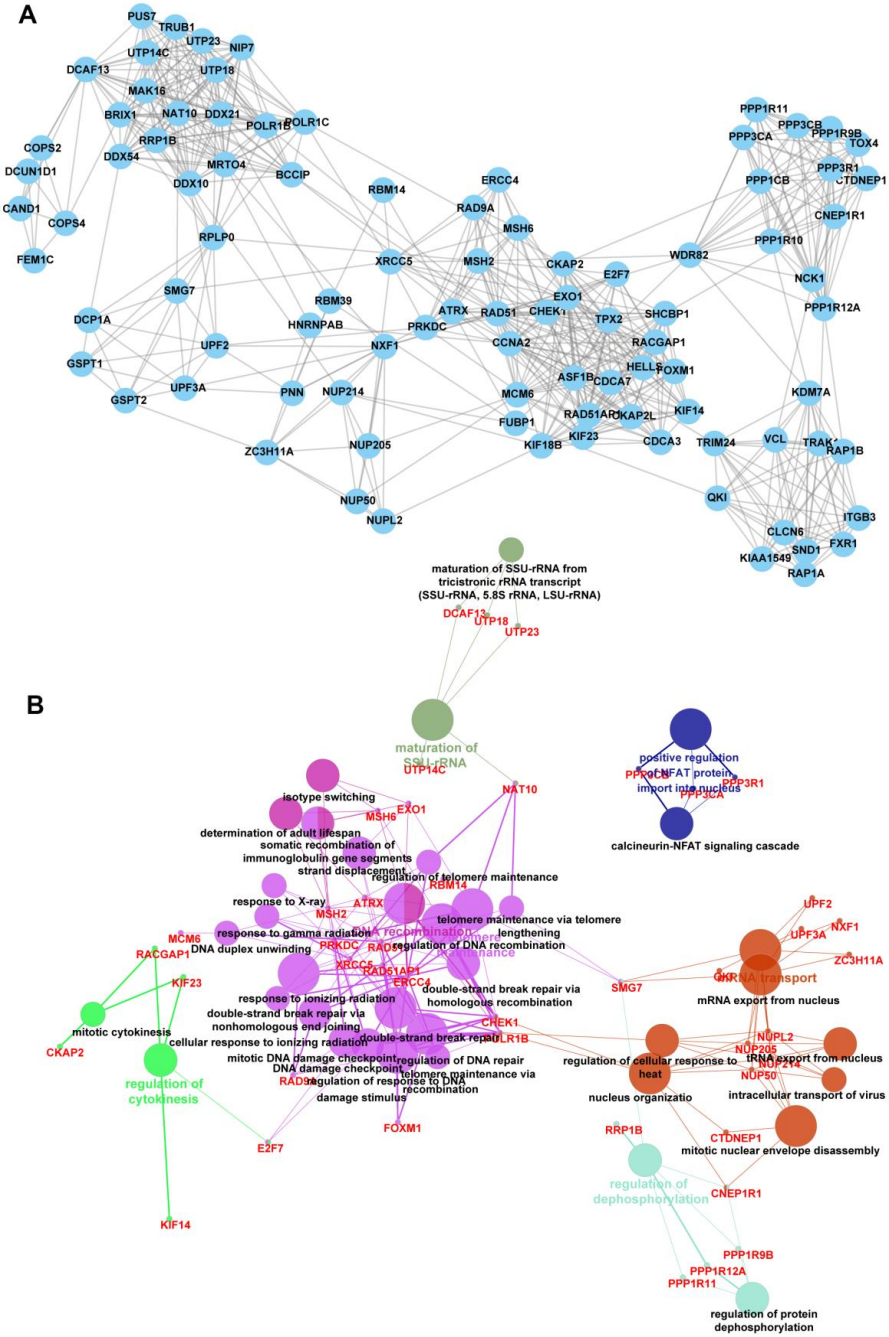
**Identification of key targets of circCCDC66-miR-320a/b axis.** (**A**) There are 93 nodes in network 3. (**B**) Bioinformatics analysis of network 2.

The network 1 included 68 nodes (Supplementary Figure 2A) (SF3B1, TRIM36, RBMX, NCBP2, TRIM41, SRSF7, UBE2C, PAPOLA, HNRNPF, AREL1,). The network 2 included 40 nodes (Supplementary Figure 3A) (STAG2, RAB5B, TFRC, CLASP1, CLIP1, SH3GL1, SKA2, EPN2, PAFAH1B1, ADRB2) and the network 3 included 93 nodes (Figure 4A) (FOXM1, ASF1B, CLCN6, MRTO4, TRAK1, PPP3CA, EXO1, TRIM24, CCNA2, NAT10).

Bioinformatics analysis showed network 1 was enriched in protein ubiquitination involved in ubiquitin-dependent protein catabolic process, fibroblast growth factor receptor signaling pathway, mRNA cis splicing, DNA-templated transcription, termination (Supplementary Figure 2B). network 2 was enriched in regulation of dendritic spine morphogenesis, Arp2/3 complex-mediated actin nucleation, sister chromatid cohesion (Supplementary Figure 3B). network 3 was involved in regulating calcineurin-NFAT signaling cascade, cellular response to ionizing radiation double-strand break repair, DNA damage checkpoint, DNA duplex unwinding, DNA recombination, double-strand break repair via nonhomologous end joining, ERCC4 double-strand break repair via, homologous recombination, maturation of SSU-rRNA, mitotic cytokinesis, mitotic DNA damage checkpoint regulation of DNA repair, mitotic nuclear envelope disassembly, mRNA export from the nucleus, mRNA transport, nucleus organization, regulation of cellular response to heat, regulation of cytokinesis, regulation of DNA recombination, regulation of protein dephosphorylation, regulation of response to DNA damage stimulus, regulation of telomere maintenance, response to gamma radiation, response to ionizing radiation, response to X-ray, somatic recombination of immunoglobulin gene segments, telomere maintenance via recombination, telomere maintenance via telomere, tRNA export from the nucleus (Figure 4B).

### CircCCDC66 could regulate FOXM1 expression by sponging miR-320a

**Figure 5:**
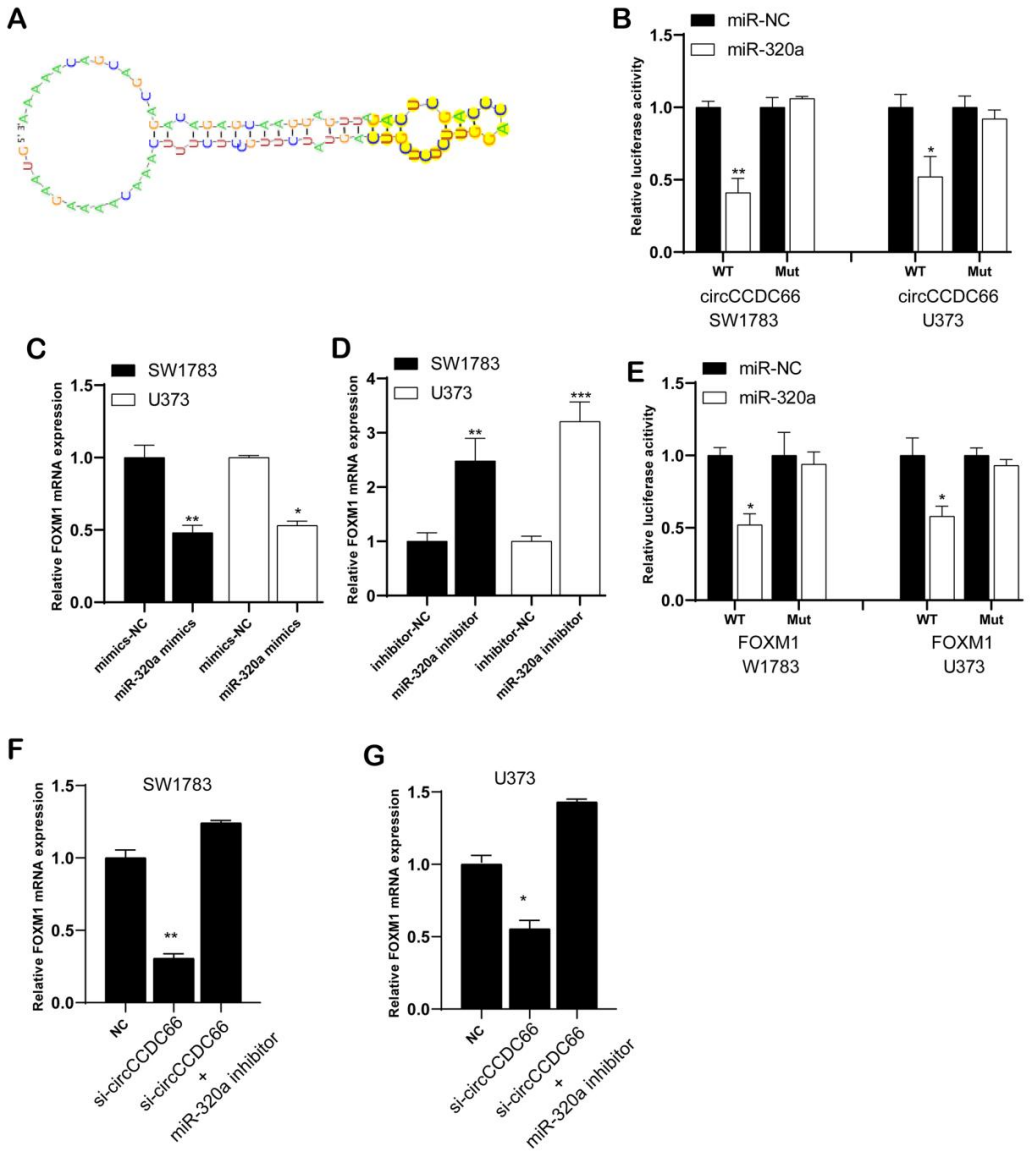
**CircCCDC66 could regulate FOXM1 expression by sponging miR-320a.** (**A**) The links between circCCDC66 and miR-320a. (**B**) Luciferase reporter assay was conducted to evaluate the interaction ability between miR-320a and circCCDC66. (**C**) Relative FOXM1 mRNA expression in glioma cells with miR-320a mimics. (**D**) Cells with miR-320a inhibitor had higher expression levels of FOXM1 mRNA. (**E**) Luciferase reporter assay was conducted to evaluate the interaction ability between miR-320a and FOXM1 3’-UTR. (**F**, **G**) MiR-320a inhibitor can resist the decreased FOXM1 mRNA expression caused by si-circCCDC66. *P < 0.05, **P < 0.01.

Previous researches have shown five discovered miRNAs could be sponged by circCCDC66. Here, our results revealed that circCCDC66 was a probable interactor with eighteen miRNAs. Based on that, miR-320a was selected as a candidate to explore the interaction with circCCDC66. Figure 5A preliminary displayed the links between circCCDC66 and miR-320a. Luciferase assay demonstrated that overexpression of miR-320a reduced the luciferase activity of circCCDC66-WT vector, however, it did not affect the luciferase activity of circCCDC66-Mut vector (Figure 5B).

Through database analysis, we found that 1864 targets were identified as miR-320a/b targets, among which FOXM1 has a higher degree. We chose FOXM1 for the next analysis. Very interestingly, we found that FOXM1 was a target of CircCCDC66/miR-320a/b and involved in regulating DNA damage response pathway. Thus, we performed bioinformatics analysis to infer probably occurred interaction between FOXM1 and miR-320a. The qRT-PCR analysis revealed miR-320a mimics lowered FOXM1 mRNA expression (Figure 5C), but the effects could be reversed by miR-320a inhibitor (Figure 5D). Luciferase assay demonstrated that overexpression of miR-320a reduced the luciferase activity of the FOXM1-WT vector, however, it did not affect the luciferase activity of FOXM1-Mut vector (Figure 5E).

**Figure 6:**
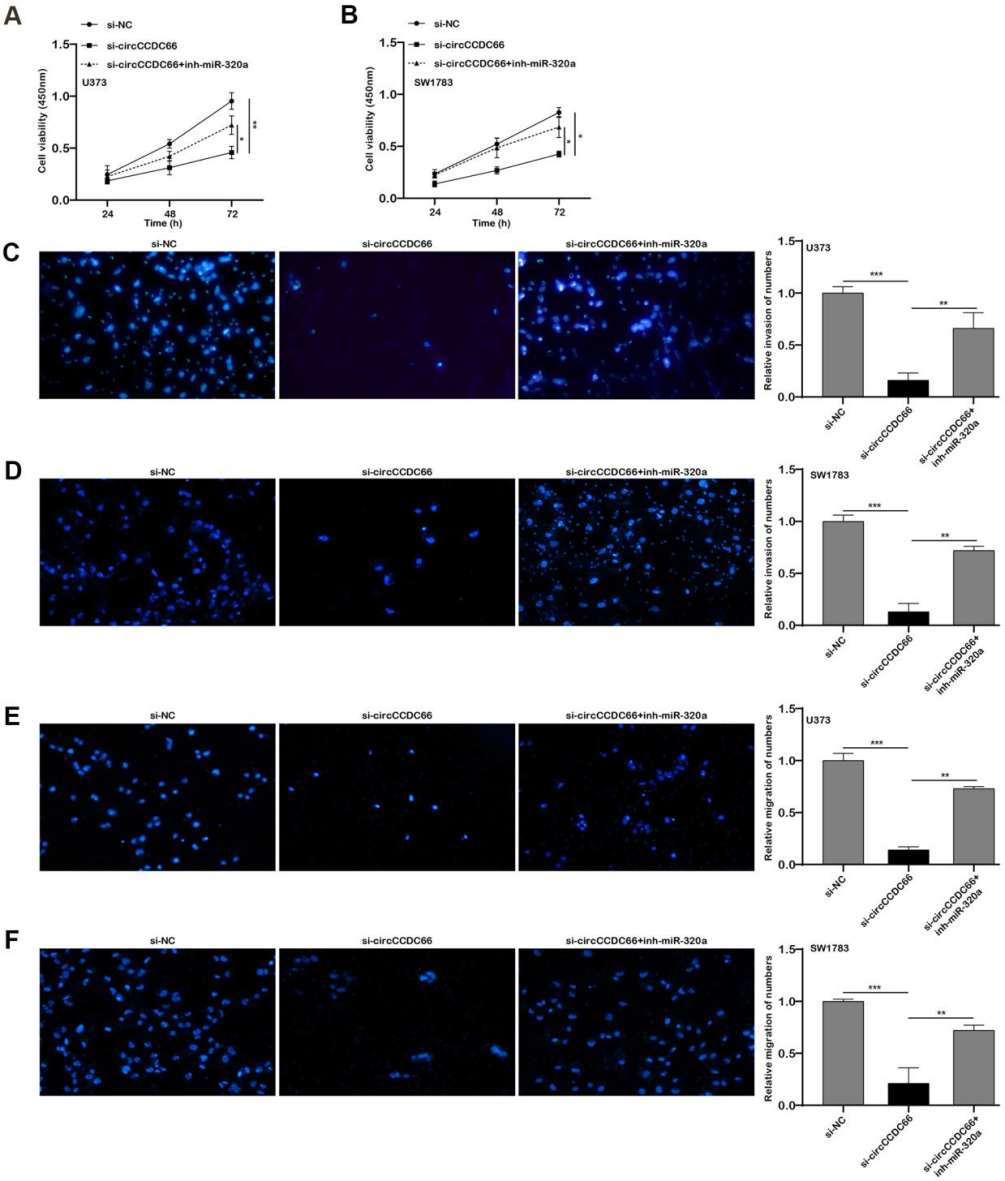
**The promoter role of circCCDC66 in SW1783 and U373 was achieved by regulating miR-320a.** (**A**, **B**) CCK-8 assays were used to evaluate cell viability. (**C**, **D**) Transwell assays were used to detect cell invasion capacities. (**E**, **F**) Transwell assays were used to detect cell migration capacities. *P < 0.05, **P < 0.01.

Further data exhibited FOXM1 mRNA level was down-regulated when ablating circCCDC66. Co-transfection of si-circCCDC66 and miR-320a inhibitor would partly reverse FOXM1 mRNA expression level, respectively in both SW1783 and U373 cells (Figure 5F, 5G). The CCK-8 assay results showed that the inhibitory effect of si-circCCDC66 on the proliferation of SW178 and U373 cells was attenuated by miR-320a inhibitor (Figure 6A, 6B). The results of the Transwell assay showed that the inhibitory effect of si-circCCDC66 on the migration and invasion of SW178 and U373 cells was attenuated by the miR-320a inhibitor (Figure 6C–6F). These data together revealed that circCCDC66 promoted cell proliferation, migration and invasion by negatively regulating the expression of miR-320a.

### CircCCDC66 promotes cell progression via FOXM1 and miR-320a pathways in glioma

**Figure 7:**
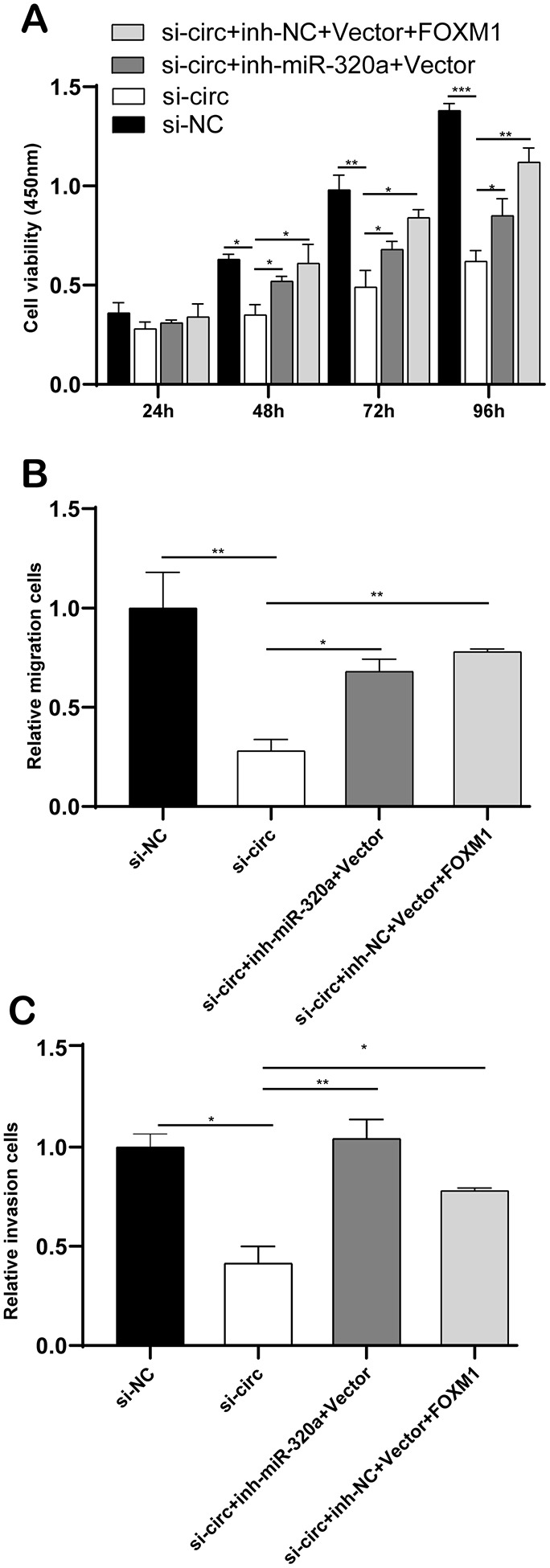
**CircCCDC66 promotes cell progression via FOXM1 and miR-320a pathways in glioma.** (**A**) CCK-8 assays were used to evaluate cell viability after transfection in U373 cells. (**B**, **C**) Transwell assays of SW13783 and U373 cells after transfection. *P < 0.05, **P < 0.01.

Figure 7A demonstrated co-transfection of inh-miR-320a or FOXM1 plasmid into U373 cells could rescue the consequence of si-circCCDC66-induced cell growth suppression to some extent. Transwell analysis revealed down-regulation of miR-320a or overexpression of FOXM1 could recover the ability of cell migration and invasion compared with the si-circ group (Figure 7B, 7C). These results suggested that circCCDC66 promotes cell progression via FOXM1 and miR-320a pathways in glioma cells.

## DISCUSSION

Glioma is the most common type of tumor in the central nervous system [24]. According to the World Health Organization (WHO) classification, gliomas can be divided into four histopathological grades (grades I-IV). Generally, we classify WHO I-II disease as low-grade glioma (LGG), and III-IV as high-grade glioma (HGG). There are significant differences in the prognosis of different grades of gliomas [25, 26]. HGG patients, especially the most malignant GBM patients, often are in poor prognostic status, with an average survival time of only 16-18 months, while patients with low-grade neoplasias can be 2 years or even up to 12 years [27]. Surgical resection is currently an important method for the treatment of gliomas [24]. Radiotherapy and chemotherapy are also required after surgery. There is also the possibility of residual tumors (especially HGG or GBM) [28]. It is necessary to find new and reliable treatments, such as molecular therapeutic targets [29]. To reveal the mechanism of glioma development, more and more researches focuses on circular RNA [30].

Currently, we have confirmed that circCCDC66 is up-regulated in glioma tissues normalized to that in normal tissues. In order to accurately find out the mechanism of action of circCCDC66, we executed experiments to validate the subcellular localization of circCCDC66 in gliomas. In order to explore the mechanisms of circCCDC66 in gliomas, we performed a bioinformatics analysis using public datasets. We constructed a circCCDC66 regulating miRNA network and revealed miR-320a was a potential target of circCCDC66, which was down-regulated in high-grade gliomas compared to low-grade gliomas. Bioinformatics analysis showed circCCDC66-miR-320a/b axis was involved in regulating multiple cancer-related pathways, such as Protein metabolism, Regulation of nucleobase, PDGF receptor signaling network, VEGF and VEGFR signaling network, EGFR-dependent Endothelin signaling events, Sphingosine 1-phosphate (S1P) pathway, Proteoglycan syndecan-mediated signaling events. PDGF receptor signaling network has been reported in prostate cancer and breast cancer. Angiogenesis disorders occur in various pathologies and are one of the hallmarks of cancer. VEGF is a key angiogenic factor. During tumor development, VEGF is enhanced pathologically. The potent pleiotropic lipid mediator sphingosine-1-phosphate (S1P) is involved in many cellular processes, including angiogenesis and cell survival, proliferation and migration. Furthermore, we identified several key targets of the circCCDC66-miR-320a/b axis. Among these, we identified FOXM1 as a key target of circCCDC66, which was involved in regulating DNA damage response pathways. In this literature, we uncovered miR-320a was one target of circCCDC66. The interaction between circCCDC66, miR-320a and FOXM1 was confirmed. These results indicate that circCCDC66 is closely related to the progression of gliomas.

Circular RNAs are derived from exonic circRNA or intronic circRNA and contain one or more miRNA binding sites. They have adsorption and inhibitory effects on miRNA and play a role in mediating miRNA targeting [31, 32]. CircBCRC-3 can directly interact with miR-182-5p and participate in bladder cancer progression [33]. In addition, circRNA_100269 was down-regulated in gastric cancer (GC) tissues, and the expression downstream of miR-630 had a negative correlation with circRNA_100269 expression [34]. The above results revealed that the circRNA_100269-miR-630 participated in the cell growth of GC. Similar to previously described, our data showed that circCCDC66 was enhanced in glioma cells. Down-regulation of circCCDC66 would result in restraint of glioma cell growth, migration, and invasion. The functional assay also indicated that circCCDC66 acted as an oncogene in glioma cells. Luciferase assay further demonstrated the interaction of circCCDC66 and miR-320a. Besides, our data also have shown that FOXM1 expression was positively correlated with the expression of circCCDC66 in glioma tissues. Analysis of the dual-luciferase reporter assay confirmed that miR-320a could bind to FOXM1 3'-UTR. FOXM1 belonged to the forkhead box transcription factor family and comprised 44 members in humans. Overexpression of FOXM1 has been detected in multiple cancer types, suggesting that FOXM1 is essential for tumorigenesis [35]. In normal cells, FOXM1 is primarily responsible for cell proliferation, while in cancer cells, FOXM1 is the hallmark of all cancers described by Hanahan and Weinberg [36]. Increasing the level of FOXM1 is related to some clinicopathological characteristics, such as tumor size, TNM stage, lymphatic metastasis, and distant metastasis [37]. Increasing the expression of FOXM1 on the carcinogenic effects of colorectal cancer through β-catenin activation signaling pathway [4]. FOXM1 may be an attractive therapeutic target and an excellent biomarker for endometrial cancer prognosis [38]. In this work, we found that circCCDC66’s promotion of cell growth was due in part to the miR-320a/FOXM1 axis.

This study also has some limitations. First, although the expression level of circCCDC66 was detected in a small sample size of glioma samples, a larger sample size should be used to further verify the correlation between the expression of circCCDC66, miR-320a and FOXM1 and clinical parameters. In addition, the protein level of FOXM1 and the expression level of circCCDC66 need to be verified in more cell models. Secondly, we will conduct gain-of-function to verify the function of circCCDC66 in glioma cells. At last, the target genes identified in this study through bioinformatics analysis that might play a key role in gliomas should also be functionally studied to further verify our findings.

In conclusion, our research firstly investigates the role of circCCDC66 in gliomas. The expression of circCCDC66 is increased in gliomas, which promotes tumor development and metastasis by regulating FOXM1 expression through miR-320a. In short, we discover a new potential therapeutic pathway: the circCCDC66-miR-320a-FOXM1 pathway.

## Supplementary Material

Supplementary Figures
