# Supplementary material for: Circular RNA circCCDC66 promotes glioma proliferation by acting as a ceRNA for miR-320a to regulate FOXM1 expression
Source: Aging (Albany NY). 2021 Jul 12;13(13):17673–89. doi: 10.18632/aging.203258 (PMC8312454; doi:10.18632/aging.203258)
Supplement: Supplementary Figures [file aging-13-203258-s001.pdf]

SUPPLEMENTARY FIGURES

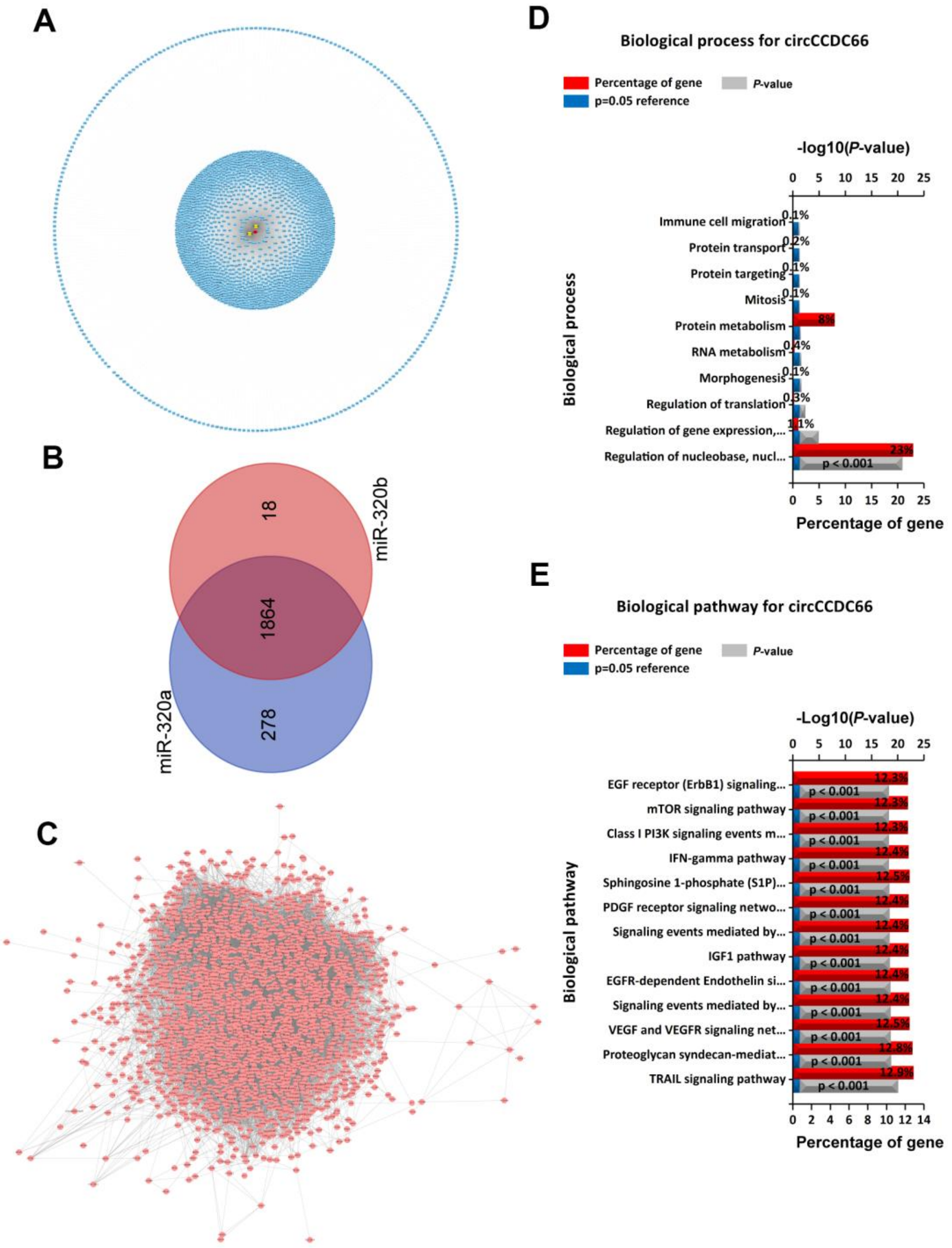

Supplementary Figure 1. Bioinformatics analysis of circCCDC66-miR-320a/b axis. (**A**) circCCDC66-miR-320a/b-mRNAs networks in gliomas using Starbase datasets. (**B**) miRNA-320a and miRNA-320b have1864 common targets. (**C**) The interaction between the proteins. (**D**) GO analysis and KEGG pathway analysis of circCCDC66. (**E**) Biological pathway for circCCDC66.

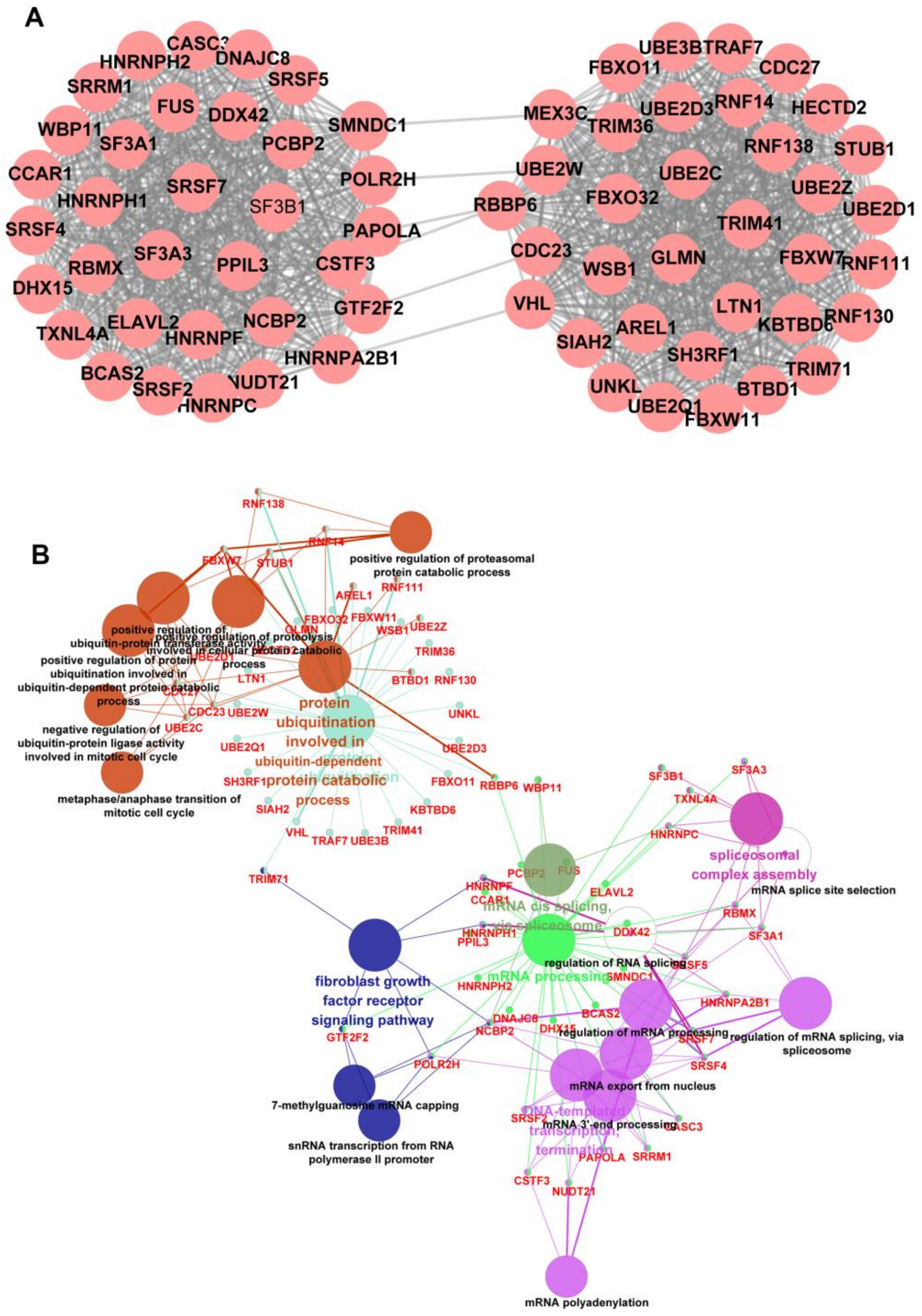

Supplementary Figure 2. Part I of identification of key targets of circCCDC66-miR-320a/b axis. (**A**) There are 68 nodes in network 1. (**B**) Bioinformatics analysis of network 1.

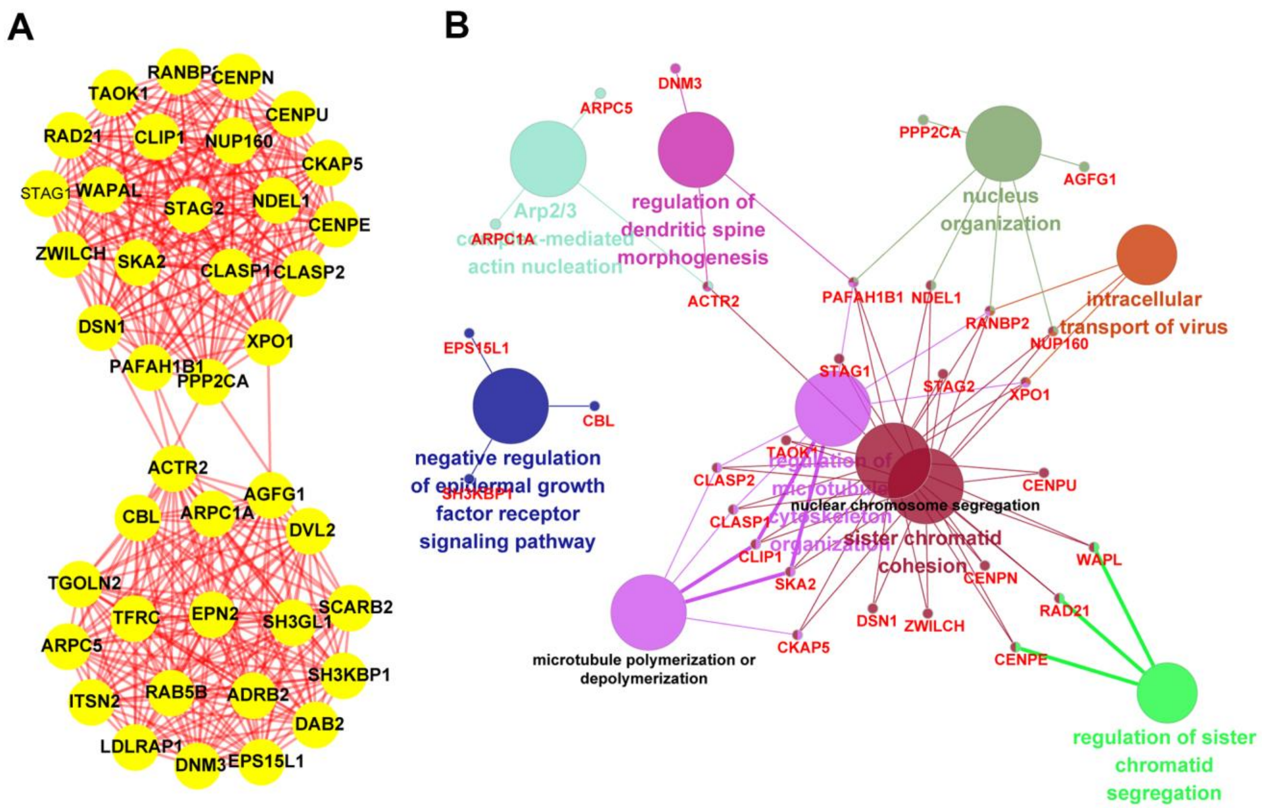

Supplementary Figure 3. Part II of identification of key targets of circCCDC66-miR-320a/b axis. (**A**) The network 2 included 40 nodes. (**B**) Bioinformatics analysis of network 2.
